# The Prevalence of Risk Factors for Cardiovascular Diseases in Najran Region, Saudi Arabia

**DOI:** 10.3390/jpm14050470

**Published:** 2024-04-29

**Authors:** Mohammad H. Alyami, Hussain Mahdi Al-Slaim, Hamad Mana Alzamanan, Mohammad F. Bayan, Khalid Ahmed

**Affiliations:** 1 Department of Pharmaceutics, College of Pharmacy, Najran University, Najran 66462, Saudi Arabia; mhalmansour@nu.edu.sa (M.H.A.); 442101197@nu.edu.sa (H.M.A.-S.); 442100157@nu.edu.sa (H.M.A.); 2 Faculty of Pharmacy, Philadelphia University, P.O. Box 1, Amman 19392, Jordan; 3 Department of Clinical Pharmacy, College of Pharmacy, Najran University, Najran 66462, Saudi Arabia; kaltigani@nu.edu.sa

**Keywords:** cardiovascular, community awareness, Saudi Arabia, Najran

## Abstract

The primary goal of this study was to investigate the knowledge, prevalence, and risk factors of cardiovascular diseases among individuals in the Najran region of Saudi Arabia. In the Najran region of Saudi Arabia, an online cross-sectional survey was conducted. Between September and October 2023, a self-administered questionnaire was distributed to a random sample of the general population aged 18 and up. The survey instrument asked about history and exposure, physician-diagnosed illnesses, cardiovascular diseases (CVDs), medication use, and other risk factors. This research had a total of 2046 individuals. Around one-fifth of the study participants reported that they or a family member suffered from CVD, and arrhythmia was the most commonly reported; blood tests, cardiac catheterization, and ECG were the most commonly reported tests performed for CVD patients, around one-tenth of CVD patients reported that they do not have any chronic diseases other than CVD, and the vast majority of the patients confirmed their regular medical appointments. This is one of the first studies to investigate the knowledge, prevalence, and use of CVD drugs among individuals in the Najran region of Saudi Arabia. The study participants’ lack of knowledge about CVD could lead to ineffective preventive measures and poor patient outcomes. The study’s findings underscore the crucial need for more extensive and efficient educational initiatives that consider the targeted population’s talents, attitudes, and perceptions.

## 1. Introduction

In both developed and developing communities, cardiovascular disease (CVD) is the primary cause of death. Heart disease, cerebrovascular disease, peripheral artery disease, rheumatic heart disease, deep vein thrombosis, and pulmonary embolism are all included in the category of CVDs [1]. Circulatory disease is sometimes used as a synonym for other terms. Coronary heart disease (CHD) is a condition affecting the blood vessels that supply the heart muscle [2,3]. Atherosclerosis is the primary cause of CVD [4]. Lipids, inflammation, and the immune system work together to contribute to the pathophysiology of atherosclerosis, which is known to be a chronic inflammatory disease [5]. Retention of altered lipoproteins in the arterial wall is a hallmark of atherosclerosis. By recruiting monocyte-derived cells, which develop into mononuclear phagocytes and consume the deposited lipoproteins to become “foam cells”, these altered lipoproteins also stimulate local macrophages [6]. Understanding CVD and the risk factors that contribute to it is essential for changing people’s health-related attitudes, behaviors, and lifestyle choices. An increase in awareness regarding the symptoms of a heart attack or stroke may enable patients to seek medical attention earlier and potentially improve their prognosis [7]. Individuals who have a thorough understanding of these factors will be better able to take proactive steps to lower their risk. According to the literature, coronary artery disease, hypertension, stroke, peripheral artery disease, and congenital heart disease were the most common CVDs in Saudi Arabia [8]. Still, most CVDs could have been avoided by managing the different risk factors linked to CVD incidence. According to the literature, there are risk factors for CVDs that can be changed, as well as ones that cannot [9]. Modifiable risk factors include, for example, those that are controlled or minimized by changing an individual’s behavior, also referred to as behavioral factors. Examples of such behavioral factors include factors related to diet (such as excessive consumption of fatty foods, sugars, and carbohydrates), smoking, alcohol consumption, physical activity, and sleeping patterns. On the other hand, nonmodifiable risk factors for CVDs include things such as age, gender, genetics and ethnicity, and family history, which are uncontrollable. Few studies have evaluated people’s knowledge of CVDs, especially among those living in the Saudi Arabian Najran region [10]. International research has been performed on the perspectives of patients and healthcare professionals. For example, healthcare professionals had good knowledge about CVDs, whereas patients with CVDs had suboptimal knowledge, diabetes patients had satisfactory knowledge, and the public had low knowledge about CVDs [11,12,13,14,15,16,17,18,19,20,21]. As a result, it is crucial to evaluate the public’s knowledge of CVD to raise awareness of the condition and avert any potential negative effects linked to CVD risk. Moreover, there are few studies conducted in Saudi Arabia on the public’s awareness of CVD risk factors and primary preventive measures. There has been no research on the understanding of many aspects of CVD among adults in Najran, Saudi Arabia. Thus, the goal of this study was to evaluate Najran citizens’ awareness of cardiovascular disease (CVD) risk factors and primary preventive measures.

## 2. Methods

A cross-sectional online poll was conducted in the Saudi Arabian Najran region. Between September and October 2022, a self-administered questionnaire was given to a random sample of the general population, aged 18 years or older. Adults aged 18 and over who live in the Najran region of Saudi Arabia were eligible to participate in this study. There were no excluding factors based on gender. Convenience sampling was the method used to collect the study sample. Participants were given the choice to continue or withdraw from the study, along with an informed consent form, on the first page of the questionnaire. The study outlined its goals in detail to make sure the patients understood the importance of taking part. The invitation letter for the study contained the inclusion criteria. Participants were only requested to take part if they satisfied the requirements for participation. To encourage others who might be interested in participating, the survey’s URL was shared on Facebook, Instagram, and Snapchat. The research instrument was created after a thorough examination of the literature. The survey instrument asked about history (family history of CVDs, kinds of CVDs, years of suffering or progression of the disease, tests performed during diagnosis, comorbidities) and exposure (whether they used tobacco, lived a healthy lifestyle, participated in sports on a regular basis (“How many days do you exercise for at least 30 min, such as walking, running, cycling, etc.?”), whether they ate fast food frequently (“Do you consider the nature of your eating to be healthy?”)). In addition, they were asked if a physician diagnosed disorders or CVD symptoms (“What types of cardiovascular problems were diagnosed?”) and what tests were performed during the diagnosis (multiple options). They were also asked whether they encountered any issues while exercising, such as breathing difficulties or chest pain (“Do you now experience any CVD symptoms?”), as well as what they tracked to avoid complications from cardiovascular disease. The questionnaire also inquired about their medication use (if they took medications for CVSs or other diseases, their adherence to medications, the kinds of drugs, and whether they used any orthodox or herbal therapy to treat any type of CVD). Participants’ understanding (potential reasons for the spread of CVD and the high-risk population affected by CVD) was also assessed. The questionnaire instrument was evaluated and verified by two clinical pharmacists from the Faculty of Pharmacy at Najran University. Through the participants' feedback, the clarity, comprehensibility, and face validity of the questions in the online questionnaire were verified, as well as whether any of them posed difficulties in terms of comprehension. Additionally, they were questioned about any inquiries that they found exasperating or offensive. They remarked on the questionnaire’s simplicity and ease of comprehension and completion. Before implementing the questionnaire on a broader scale, a preliminary online study was conducted with a small sample of the general population (40 participants) to assess comprehension. The results verified that the questionnaire was straightforward and easily comprehensible.

The minimum required sample size was 385 individuals using a 95% confidence interval, a standard deviation (SD) of 0.5, and a 5% margin of error. The data from this study were analyzed using the R STAT software (version 4.2.1). The data for this investigation are presented using descriptive statistics. The categorical variables are displayed as frequencies and percentages. The Chi-Square test of independence was employed to assess the statistical disparity between participants with CVDs and those without with regard to their demographic variables. CVDs were analyzed using binary logistic regression to determine risk variables. At *p* < 0.05, statistical significance was deemed acceptable. The results of descriptive statistics were reported as frequencies (percentages). The respondents’ knowledge of the following was assessed using a scoring system: eleven CVD risk factors, five warning signs for heart attacks, five warning signs for strokes, and six forms of CVD. The following categories applied to the knowledge scores for each section: knowledge of CVD types is divided into three categories: low (≤2), moderate (3), and high (≥4). Knowledge of heart attack or stroke warning symptoms is divided into three categories: low (≤2), moderate (3), and high (≥4). Knowledge of CVD risk factors is divided into three categories: low (≤5), moderate (6–8), and high (≥9). Ultimately, the respondent’s scores for CVD types, heart attack symptoms, stroke symptoms, and CVD risk factors were added up to determine the overall CVD knowledge score, which was then computed as a continuous variable. Low knowledge (≤10), moderate knowledge (11–16), and excellent knowledge (≥17) were assessed, with a maximum total knowledge score of 21. To ascertain how each independent variable related to general knowledge of CVD, univariate logistic regression was used. To identify the variables that are independently linked with overall CVD knowledge, the multiple logistic regression analysis included all variables with *p* ≤ 0.25 in the univariate study. Only the odds ratio (OR) and 95% confidence interval from the multivariate logistic analysis are presented. At *p* < 0.05, statistical significance was deemed acceptable. Response choices for the dependent variable in each model were divided into two categories: “low knowledge” and “moderate/high knowledge”.

## 3. Results

Table 1 shows the demographic and clinical characteristics of the study participants. A total of 2046 individuals agreed to be included in the study, and their median (IQR) age was 28 years. Of the respondents, 63.5% were males, 74.2% had higher education, and 52.8% had a low monthly income of less than 5000 Saudi Riyals. In addition, 69.1% of respondents indicated that they are single, 29.8% are employed, and 49.3% of the employed individuals reported office-based work only. Table 2 shows the respondents’ CVD risk factors. Four hundred and twenty-five participants (20.8%) reported that they smoked. Approximately one-tenth (9.9%) of the respondents indicated that they have chronic disease, 17.5% considered themselves at risk of CVD, and 73.1% indicated that they have trouble taking their medicine as directed. A total of 23.1% reported that they are obese, 55.1% indicated that they are free of stress, 38.3% indicated that they used to exercise for 30 min 3–5 times weekly, and 37.0% reported eating healthy food. Table 3 shows the respondents’ CVD history profile. A total of 414 persons (20.2%) have a family history of CVD, and 17.8% confirmed diagnosis. Arrhythmia (5.7%), heart valve diseases (5.3%), and cardiovascular diseases (5.0%) were the most common types of CVDs. Blood tests (11.1%), cardiac catheterization (8.7%), and ECG (7.8%) were the most common types of investigations. A total of 146 patients (7.1%) were diagnosed more than 5 years ago, and 48 (2.3%) were diagnosed at birth. In addition, 5.4% of the patients reported that they have suffered from sweating, 6.7% reported chest pain, 6.5% stated feeling extremely anxious, 6.8% felt dizzy, 3.5% felt sick, 5.1% reported shortness of breath, coughing, or wheezing, and 7.3% felt pain elsewhere in the body, such as the arms, neck, or back. A total of 173 patients confirmed their suffering from lack of physical activity, and 5.5% indicated their family history of heart disease, while 215 patients indicated that they do not have any chronic disease other than CVD. In addition, 249 cases confirmed having regular medical appointments, and 161 and 141 patients followed medical treatments and healthy lifestyles, respectively, to avoid complications of CVD. Table 4 shows the respondents’ knowledge of CVD types and heart attack and stroke symptoms. The majority of the participants (47.2%) believe that men are more susceptible to CVD than women and children, and 17.5% are not sure who is more susceptible. The most common heart attack symptom reported was “chest pain or discomfort” (47.7%), followed by “difficulty breathing or shortness of breath” (46.1%) and “feeling weak, dizzy, or fainting” (41.9%). The most common stroke symptoms indicated by participants were “sudden dizziness, difficulty walking, or loss of balance or coordination” (40.7%), followed by “sudden numbness or weakness in the face, arm, or leg” (38.4%) and “sudden difficulty seeing in one or both eyes” (35.8%). The most common risk factors identified by respondents were smoking, obesity, genetics, and stressful lifestyles. The most common type of CVD identified was cardiovascular disease (35.4%), followed by heart valve disease (32.1%), arrhythmia (28.8%), and heart failure (27.8%). Table 5 shows the binary logistic regression analysis. At *p* < 0.05, statistical significance was deemed acceptable. Response choices for the dependent variable in each model were divided into two categories: “low knowledge” and “moderate/high knowledge”.

## 4. Discussion

Cardiovascular disease (CVD) is widely recognized as the leading cause of death globally, with over 80% of all CVD-related mortality rates occurring in low-income and Middle Eastern nations [11]. However, because the majority of CVD risk factors are under individuals' control, it is seen as a preventable disease. This research is one of the first few studies conducted in the Najran region of Saudi Arabia on the public’s awareness of CVD risk factors and primary preventive measures. Modifying people’s health-related attitudes, behaviors, and lifestyle choices requires an understanding of CVD and the risk factors that lead to it. An improved understanding of the signs of a heart attack or stroke could help patients seek treatment sooner and possibly lead to a better outcome. People will be more equipped to reduce their risk if they have a solid awareness of these issues. This study set out to assess the general public’s knowledge of the main preventive interventions and risk factors for cardiovascular disease (CVD) in Najran. This study’s strength is that we generated representative data on the study population by using an adequate sample size and sampling technique. The high response rate is another asset. Thus, the findings can be applied to Najran’s population as a whole. There are drawbacks to this kind of research, which employed a self-administered questionnaire. It is highly dependent on the information provided by respondents and susceptible to memory bias or inaccuracy. This kind of survey, which was taken at face value, makes it impossible to determine the degree of true responses or to independently check respondents’ assertions. The cross-sectional nature of the data, which only reflected one point in time and could not account for changes in respondents’ knowledge of CVDs, is another drawback of the study. The median (IQR) age was 28 years. This young age can be attributed to the contact method used in this study (social media) and to the fact that the majority of the population in Saudi Arabia is young (63% under the age of 30). The key findings are the following: (1) around one-fifth of the study participants reported that either themselves or one of their family members suffered from CVD, and arrhythmia was the most commonly reported; (2) blood tests, cardiac catheterization, and ECG were the most commonly reported tests performed for CVD patients; (3) around one-tenth of CVD patients reported that they did not have any chronic disease other than CVD; (4) the majority of them reported that they followed medical treatments and healthy lifestyles to avoid complications of CVD; (5) the vast majority of the patients confirmed having regular medical appointments; (6) the majority of the participants believed that men are more susceptible to CVD than women and children; (7) participants who were obese, aged more than 40 years, and did not have a healthy lifestyle were more likely to have CVD than others. The present study’s highly concerning discovery was how little the respondents knew about the warning signs of a heart attack or stroke. “Chest pain” and “sudden dizziness, difficulty walking, or loss of balance or coordination” were the most often reported symptoms. These findings highlight the critical need to educate the Najran community on the various symptoms of heart attacks and strokes, since improved understanding might result in early presentation for medical care, thus improving patient outcomes. Respondents’ knowledge of CVD risk factors was also low. Only around one-third of respondents identified nine risk factors. These findings underscore the urgent need to educate the Najran community on the numerous CVD risk factors and to raise public knowledge of CVD risk factors and primary preventative strategies. In our study, around one-fifth of the study participants reported that either themselves or one of their family members suffered from CVD. Arrhythmia was the most commonly reported. This confirmed the findings of previous studies performed in different areas throughout the world [12,13,14,15,16,17]. In the current survey, age, type of work, monthly income, obesity, and eating a healthy diet were found to be significantly associated with CVD knowledge (*p* < 0.05). Those in the 41–50 and 51–60 age groups knew more about CVD than people in other age groups according to the results of the current study. Prior research findings documented a noteworthy correlation between age and understanding of CVD [18,19]. This is in contrast to other research that found no discernible variation in the knowledge of CVD among age groups [19,20,21]. It is possible that schools in Najran lack effective and appropriate CVD education programs given the low levels of awareness among younger age groups. They will probably accumulate information from the media as the years pass. Participants in the study who said that they followed a healthy diet every day knew more about CVD. This is in line with research from literature showing that people with more awareness of their food had better knowledge of CVD. The present findings additionally show a strong correlation between the respondents’ awareness of CVD and their type of employment, monthly income, and obesity. This fits with other research that found that people with high BMI and low income had poor knowledge [14,15,16,17,18].

## 5. Conclusions

This study aimed to show the general Najran population’s baseline knowledge of CVD risk factors, the warning signs of a heart attack or stroke, and the forms of CVD. The study participants’ inadequate understanding of CVD could result in inadequate preventative actions and less-than-ideal patient outcomes. The results of this study highlight the critical need for more widespread and efficient educational interventions that consider the talents, attitudes, and perceptions of the targeted population.

## Figures and Tables

**Table 1:** Demographic and clinical characteristics of the study participants (n = 2046).

Characteristics	Frequency (%)
**Nationality**	
Saudi	1815 (88.7)
Non-Saudi	231 (11.3)
**Gender**	
Male	1300 (63.5)
Female	746 (36.5)
**Marital Status**	
Single ^1^	1414 (69.1)
Married	632 (30.9)
**Age (Years)**	
18–30	1250 (61.1)
31–40	355 (17.4)
41–50	293 (14.3)
51–60	121 (5.9)
>60	27 (1.3)
**Labor Force**	
Unemployed ^2^	1437 (70.2)
Employed	609 (29.8)
**Types of Employment**	
Office-based	1008 (49.3)
Field	437 (21.4)
Both (office-based and field)	601 (29.3)
**Education Level**	
Low education level ^3^	527 (25.8)
High education level ^4^	1519 (74.2)
**Residence**	
Najran	1463 (71.5)
Khobash	123 (6.0)
Habona	192 (9.4)
Beir Askar	39 (1.9)
Badr Alganob	92 (4.5)
Almentasher	51 (2.5)
Thar	86 (4.2)
**Monthly Income**	
<5000 Saudi Riyals	1081 (52.8)
5000–10,000 Saudi Riyals	327 (16.0)
10,000–15,000 Saudi Riyals	317 (15.5)
15000–20,000 Saudi Riyals	189 (9.2)
>20,000 Saudi Riyals	132 (6.5)

^1^ Including single, divorced, and widowed. ^2^ Including unemployed, students, and freelancers. ^3^ Including school levels (primary, secondary, and high school). ^4^ Including diploma, bachelor, and postgraduates.

**Table 2:** Respondents’ CVD risk factors (n = 2046).

Characteristics	Frequency (%)
**Smoking**	
No	1621 (79.2)
Yes	425 (20.8)
**Chronic Disease**	
No	1864 (91.1)
Yes	182 (9.9)
**Drug Adherence**	
No	1495 (73.1)
Yes	337 (16.5)
Sometimes	214 (10.4)
**Self-reported Weight**	
Non-obese	1573 (76.9)
Obese	473 (23.1)
**Lifestyle**	
Very stressful or stressful	269 (13.1)
Relative stressful	646 (31.6)
Free of stress	1131 (55.3)
**Exercising for 30 min/Week**	
0–1	1026 (50.1)
3–5	783 (8.3)
>5 times	237 (11.6)
**Healthy Eating**	
No	1288 (63.0)
Yes	758 (37.0)
**Do you consider yourself at risk of CVD?**	
No	905 (44.2)
Yes	357 (17.5)
Not sure	784 (38.3)

**Table 3:** Respondents’ CVD history profile (n = 2046).

Characteristic	Frequency (%)
**Family History of CVD**	
No	1632 (79.8)
Yes	414 (20.2)
**Types of Diagnoses**	
Unconfirmed	49 (2.4)
Confirmed	365 (17.8)
**Types of CVD**	
Cardiovascular diseases	103 (5.0)
Arrhythmia	117 (5.7)
Diseases of congenital heart defects	65 (3.2)
Cardiomyopathy	84 (4.1)
Heart diseases caused by infections of the heart membranes	32 (1.6)
Heart valve diseases	109 (5.3)
Cardiac arrest	38 (1.9)
Coronary heart disease	31 (1.5)
Angina	33 (1.6)
Heart attack	23 (1.1)
Heart failure	29 (1.4)
Hypertension	80 (3.9)
Atrial fibrillation	20 (1.0)
Arteriosclerosis	58 (2.8)
Septal defect	35 (1.7)
**Tests and Investigations**	
Blood test	228 (11.1)
X-ray	105 (5.1)
ECG	160 (7.8)
Monitoring of the heart with a Holter device	86 (4.2)
Echocardiography	132 (6.5)
Transesophageal echocardiogram (TEE)	63 (3.1)
Cardiac catheterization	178 (8.7)
Cardiac biopsy	41 (2.0)
CT scan	90 (4.4)
MRI	80 (3.9)
**Time Since Diagnosis**	
1 Year	63 (3.1)
2 Years	63 (3.1)
3 Years	61 (3.0)
4 Years	33 (1.6)
More than 5 Years	146 (7.1)
At birth	48 (2.3)
**Symptoms**	
Sweating	110 (5.4)
Chest pain	137 (6.7)
Feeling extremely anxious	134 (6.5)
Feeling dizzy	139 (6.8)
Feeling sick	71 (3.5)
Shortness of breath, coughing, or wheezing	105 (5.1)
Pain elsewhere in the body, such as arms, neck, or back	150 (7.3)
**Risk Factors**	
Tobacco use (including vaping)	82 (4.0)
High cholesterol (hyperlipidemia)	77 (3.8)
Family history of heart disease	112 (5.5)
Lack of physical activity	173 (8.5)
Overweight or obesity	98 (4.8)
Having diabetes	88 (4.3)
Hypertension	77 (3.8)
Alcohol abuse	14 (0.7)
**Chronic Disease other than CVD**	
No	215 (10.5)
Chronic dermatological disease	29 (1.4)
Endocrine and diabetes	68 (3.3)
Respiratory disease	41 (2.0)
Rheumatoid and immune disease	44 (2.2)
Any type of hypersensitivity	32 (1.6)
Renal transplant	39 (1.9)
Alzheimer’s disease	28 (1.4)
Diabetes and hypertension	51 (2.5)
Diabetes	35 (1.7)
Colon disease	48 (2.3)
Renal disease	14 (0.7)
Epilepsy	13 (0.6)
**Preventive/Treatment Strategies**	
Medical treatments	161 (7.9)
Surgical treatment	77 (3.8)
Vitamins and supplements	109 (5.3)
Healthy lifestyle	141 (6.9)
None of the above	107 (5.2)
**Regular Checkups**	
No	165 (8.1)
Yes	249 (12.2)

**Table 4:** Respondents’ knowledge of CVD types and heart attack and stroke symptoms (n = 2046).

Characteristic	Frequency (%)
**Who is more susceptible to CVD?**	
Men	965 (47.2)
Women	496 (24.2)
Children	228 (11.1)
Not sure	357 (17.5)
**Symptoms of cardiac attack?**	
Pain or discomfort in the jaw, neck, or back	471 (23.0)
Feeling weak, dizzy, or fainting	857 (41.9)
Chest pain or discomfort	975 (47.7)
Pain or discomfort in the arms or shoulder	495 (24.2)
Difficulty breathing or shortness of breath	943 (46.1)
**Symptoms of stroke?**	
Sudden numbness or weakness in the face, arm, or leg	785 (38.4)
Sudden confusion or difficulty speaking or understanding others	705 (34.5)
Sudden difficulty seeing in one or both eyes	733 (35.8)
Sudden dizziness, difficulty walking, or loss of balance or coordination	832 (40.7)
Severe headache with no known cause	505 (24.7)
**Causes of CVD?**	
Obesity	879 (43.0)
Smoking	978 (47.8)
Diabetes	596 (29.1)
Genetics	718 (35.1)
Aging	604 (29.5)
Hypertension	631 (30.8)
Stressful lifestyle	668 (32.6)
Hyperlipidemia	627 (30.6)
Less physical activities	651 (31.8)
Family history	523 (25.6)
Unhealthy food	631 (30.8)
**Most common types of CVD?**	
Cardiovascular diseases	724 (35.4)
Arrhythmia	589 (28.8)
Diseases of congenital heart defects	401 (19.6)
Cardiomyopathy	526 (25.7)
Heart diseases caused by infections of the heart membranes	317 (15.5)
Heart valve diseases	657 (32.1)
Cardiac arrest	425 (20.8)
Coronary heart disease	307 (15.0)
Angina	407 (19.9)
Heart attack	500 (24.4)
Heart failure	569 (27.8)
Hypertension	495 (24.2)
Atrial fibrillation	147 (7.2)
Diabetes	518 (25.3)
Others	848 (41.4)

**Table 5:** Binary logistic regression analysis.

Characteristics	Moderate/High Frequency (%)	OR (95% CI)	*p*-Value
**Gender**			
Male	250 (19.2)	Reference	
Female	182 (24.4)	1.02 (0.99–1.06)	0.141
**Marital status**			
Single	316 (22.3)	0.95 (0.91–1)	0.0628
Married	116 (18.4)	Reference	
**Age (Years)**			
18–30	323 (25.8)	Reference	
31–40	72 (20.3)	0.96 (0.90–1.02)	0.177
41–50	29 (9.9)	0.89 (0.83–0.95)	0.004
51–60	7 (5.8)	0.86 (0.79–0.93)	0.004
>60	1 (3.7)	0.86 (0.73–1.01)	0.08
**Labor Force**			
Unemployed	102 (16.7)	Reference	
Employed	330 (23)	1.02 (0.97–1.07)	0.5
**Types of Employment**			
Office-based	264 (26.2)	Reference	
Field	47 (10.8)	0.91 (0.86–0.95)	<0.001
Both (office-based and field)	121 (20.1)	0.96 (0.92–1.0)	0.03
**Education Level**			
Low education level	117 (22.2)	Reference	
High education level	315 (20.7)	1.04 (0.99–1.08)	0.11
**Monthly Income**			
<5000 Saudi Riyals	293 (27.1)	Reference	
5000–10,000 Saudi Riyals	61 (18.7)	0.95 (0.90–1.01)	0.09
10,000–15,000 Saudi Riyals	37 (11.7)	0.92 (0.86–0.99)	0.02
15000–20,000 Saudi Riyals	22 (11.6)	0.93 (0.86–1.00)	0.04
>20,000 Saudi Riyals	19 (14.4)	0.94 (0.86–1.02)	0.12
**Self-Reported Weight**			
Non-obese	332 (19.7)	Reference	
Obese	100 (27.5)	1.08 (1.04–1.14)	<0.001
**Smoking**			
No	369 (22.8)	Reference	
Yes	63 (14.8)	0.96 (0.92–1.00)	0.08
**Exercising for 30 min/Week**			
0–1	235 (22.9)	Reference	
3–5 times	149 (19)	1.00 (0.96–1.03)	0.82
>5	48 (20.3)	1.01 (0.96–1.07)	0.69
**Healthy Eating**			
No	333 (25.9)	Reference	
Yes	99 (13.1)	0.92 (0.88–0.95)	<0.001
**Lifestyle**			
Very stressful or stressful	40 (14.9)	0.98 (0.92–1.03)	0.41
Relative stressful	136 (21.1)	1.00 (0.96–1.04)	0.99
Free of stress	256 (22.6)	Reference	
**Family History of CVD**			
No	349 (21.4)	Reference	
Yes	83 (20)	0.97 (0.93–1.02)	0.24
**Chronic Disease**			
No	397 (21.3)	Reference	
Yes	35 (19.2)	0.99 (0.93–1.06)	0.84

## Data Availability

Data are contained within the article.
